# HER2 overexpression/amplification status in colorectal cancer: a comparison between immunohistochemistry and fluorescence in situ hybridization using five different immunohistochemical scoring criteria

**DOI:** 10.1007/s00432-022-04230-8

**Published:** 2022-08-26

**Authors:** Qi Sun, Qi Li, Fuping Gao, Hongyan Wu, Yao Fu, Jun Yang, Xiangshan Fan, Xiaobin Cui, Xiaohong Pu

**Affiliations:** 1 Department of Pathology, Nanjing Drum Tower Hospital, The Affiliated Hospital of Nanjing University Medical School, 321 Zhong-Shan Road, Nanjing, 210008 Jiangsu China; 2 Center for Digestive Medicine, Nanjing Drum Tower Hospital, The Affiliated Hospital of Nanjing University Medical School, Nanjing, 210008 Jiangsu China; 3 Department of Pathology, Gaochun People’s Hospital, Nanjing, 210008 Jiangsu China

**Keywords:** Colorectal carcinomas, HER2, Fluorescence in situ hybridization, Immunohistochemistry

## Abstract

**Objective:**

Although HER2 has gradually become an important therapeutic target for colorectal cancer (CRC), a unified and standard HER2 scoring system was still not established in CRC, and the debatable results of immunohistochemistry and fluorescence in situ hybridization (FISH) in CRC requires further exploration.

**Methods:**

In this study, we use five immunohistochemical (IHC) scoring criteria (i.e., IRS-p, IRS-m, GEA-s, GEA-b and HERACLES) and two FISH criteria to evaluate HER2 status, and further evaluate the correlation between HER2 status and clinicopathological features, survival in a large, unselected Chinese cohort of 664 CRCs.

**Results:**

Finally, we set HER2/CEP17 ratio ≥ 2.0, or an average HER2 copy number ≥ 6.0 as FISH-positive threshold and the amplification rate of HER2 gene was 7.08% (47/664).The HER2 positivity (IHC 3+) was 2.71%, 3.16%, 2.56%, 2.71% and 3.16%, according to the IHC scoring criteria of IRS-p, IRS-m, GEA-s, GEA-b and HERACLES, respectively. Set FISH results as the golden standard; receiver-operating characteristic analysis showed that IRS-p had both high sensitivity and specificity than other IHC scoring systems to evaluate HER2 status. Based on IRS-p criterion, There were significant differences in tumor differentiation (*p* = 0.038), lymphatic vascular invasion (*p* = 0.001), pN stage (*p* value = 0.043), and overall survival (*p* < 0.001) among IHC score 0–3 + groups. Meanwhile, there were significant differences in pT stage (*p* = 0.031), pN stage (*p* = 0.009) and overall survival (*p* < 0.001) among FISH subgroups.

**Conclusion:**

The IRS-p criterion was more suitable for assessing the HER2 status in CRC patients than other IHC criteria. Whereas for FISH scoring system, only HER2/CEP17 < 2.0, meanwhile HER2cn < 4.0 and HER2cn ≥ 6.0 were subgroups with unique clinicopathological characteristics.

## Introduction

Colorectal cancer (CRC) is the third most frequent and the second most fatal cancer worldwide (Sung et al. 2021). Although significant progress has been made in the diagnosis and treatment of CRC in recent years, the 5-year overall survival (OS) rate of patients with CRC of all stages is only 64%. However, patients with metastatic CRC (mCRC) have a worse prognosis, with a 5-year OS rate of only 12% (Bali et al. 2021). Understanding the mechanism of genetic variations of CRC is of great significance for preventing and treating CRC.

The human epidermal growth factor receptor (HER) family, which includes epidermal growth factor receptor (EGFR, HER1, erbB1), HER2 (erbB2, neu), HER3 (erbB3), and HER4 (erbB4), has been reported to be involved in the regulation of proliferation and differentiation in a variety of tumors, especially CRC (Sergina and Moasser 2007). Genetic abnormalities in this family often lead to tumorigenesis (Mendelsohn and Baselga 2006). EGFR is an important prognostic marker and therapeutic target for CRC, which is positively expressed in 59% to 84% of CRC specimens (Rego et al. 2010; Lee et al. 2011). Its overexpression was closely related to higher clinical stage and worse disease-free survival and OS in CRC patients (Rego et al. 2010). Furthermore, the cancer genome atlas (TCGA) project identified that 7% of CRC patients harbored HER2 gene amplification or somatic mutations (Cancer Genome Atlas N 2012). HER2 protein membranous overexpression was found in 1.6–11% of patients with primary CRC (Ingold Heppner et al. 2014; Seo et al. 2014; Kavanagh et al. 2009) and 2–9.5% of mCRC patients (Ross et al. 2018; Wang et al. 2020). HER2 gene alterations (including overexpression/amplification and activating mutations) are among the most common genomic abnormalities in RAS and BRAF wild-type mCRC patients with primary resistance to anti-EGFR monoclonal antibody therapy (Cremolini et al. 2017). Therefore, HER2 is considered as an emerging therapeutic target for CRC patients (Sawada et al. 2018).

In contrast to gastric and breast cancers, the diagnostic criteria for HER2 positivity in CRC have not been fully standardized and have varied across studies (Kapitanovic et al. 1997; Richman et al. 2016; McKay et al. 2002; Park et al. 2018; Conradi et al. 2013). The HER2 amplification for colorectal cancer enhanced stratification (HERACLES) trial was a multicenter, open-label Phase II clinical trial in patients with CRC resistant to chemotherapy and anti-EGFR therapy. This trial enrolled mCRC patients with wild-type KRAS and HER2 overexpression, who were then treated with a combination of trastuzumab and lapatinib, with an objective response rate of 30% (Valtorta et al. 2015). They defined three conditions of HER2 positivity as follows (HERACLES criteria): (1) a HER2 immunohistochemical (IHC) 3 + score in ≥ 50% of CRC cells; (2) a HER2 IHC 3 + score in 10–50% of the CRC cells, and a fluorescence in situ hybridization (FISH) HER2/CEP17 ≥ 2.0 in ≥ 50% of CRC cells; (3) more than 50% of CRC cells with a HER2 IHC 2 + score and a FISH HER2/CEP17 ≥ 2.0. However, to date, HER2 diagnostic criteria for gastroesophageal adenocarcinoma (GEA criteria) (Richman et al. 2016) or other independent HER2 IHC scoring systems (Shabbir et al. 2016; Moussa et al. 2020) have also been used in CRC HER2 studies. Moreover, the prognostic role of HER2 in CRC remains controversial. Some studies have shown that HER2 overexpression/amplification, as an adverse prognostic factor, is closely correlated with the tumor stage and survival in CRC patients (Ingold Heppner et al. 2014; Kapitanovic et al. 1997; Osako et al. 1998). However, other studies have shown no association between HER2 expression and patient survival (Kavanagh et al. 2009; Richman et al. 2016; Marx et al. 2010). These controversial results suggest that the role of HER2 in CRC needs to be further explored.

In this study, we first confirmed FISH threshold, then set FISH as the golden standard for HER2 amplification to compare the performance of the five IHC scoring methods, so as to determine the most suitable IHC criteria for the evaluation of HER2 status in CRC. Meanwhile, the correlation between HER2 status and clinicopathological features and survival were also explored.

## Materials and methods

### Patients

We established an unselected cohort of CRC patients undergoing surgical resection by retrieving all cases from January 2015 to December 2019 in the computerized database of the Department of Pathology, Nanjing Drum Tower Hospital, Nanjing, China. The inclusion criteria were as follows: (1) colorectal adenocarcinoma confirmed by pathology; (2) available pathological tissue samples. Exclusion criteria included: (1) primary tumor with extracolonic or appendiceal location; (2) presence of simultaneous cancer; (3) preoperative neoadjuvant chemotherapy, radiation therapy, or immunotherapy; (4) insufficient clinicopathological data.

All tumors were histopathologically diagnosed according to the 5th edition of WHO digestive system tumors classification (Nagtegaal and Salto-Tellez 2019) and were staged following the rules specified in the 8th edition cancer staging manual of the American Joint Cancer Committee (AJCC) (Jessup et al. 2016). Primary tumors originated from cecum to transverse colon were defined as the right-sided group, and tumors located at or distal to the splenic flexure were defined as the left-sided group. Patients' consent for surgical resection and clinical research was obtained in all cases before the surgical resection. OS was calculated from the date of surgery until the last follow-up or mortality date. Follow-up information was conducted via telephone interview and medical record review. The Medical Ethics Committee gave ethics approval for this study at Nanjing Drum Tower Hospital.

### Tissue microarray construction

Each tissue sample was immediately fixed in 10% neutral buffered formalin for 12–48 h, then paraffin-embedded. Sections were deparaffinized routinely, rehydrated, and retrieved. The Grand Master automated arrayer (3DHISTECH Ltd., Budapest, Hungary) was used to create the tissue microarray (TMA) with a 2 mm punch size from representative tumor blocks for each case. Two tumor cores were punched out from each case, one was selected from the tumor center, the other was derived from the infiltrative front of the deepest tumor invasion portion, and the necrotic areas were avoided. Each TMA block contained 60 tumor cores, which were then cut into 4-μm-thick sections for HE and IHC staining, and FISH detection.

### IHC staining

HER2 IHC staining was carried out on the automatic Ventana Bench Mark Ultra system (Roche Diagnostics, Basel, Switzerland) using an automated staining protocol validated for the anti-HER2/neu monoclonal antibody (Clone: 4B5, pre-dilution, Roche Diagnostics, Basel, Switzerland). Monoclonal antibodies against MLH1 (Clone: ES05, dilution 1:100, Dako Denmark A/S, Denmark), PMS2 (Clone: EP51, dilution 1:100, Dako Denmark A/S, Denmark, Dako Denmark A/S, Denmark), MSH2 (Clone: FE11, dilution 1:100, Dako Denmark A/S, Denmark), and MSH6 (Clone: EP49, dilution 1:150, Dako Denmark A/S, Denmark) were performed according to the method previously described (Fu et al. 2021). Both positive and negative (without the primary antibody) controls were used in each run of staining.

### HER2 IHC scoring method

**Fig. 1:**
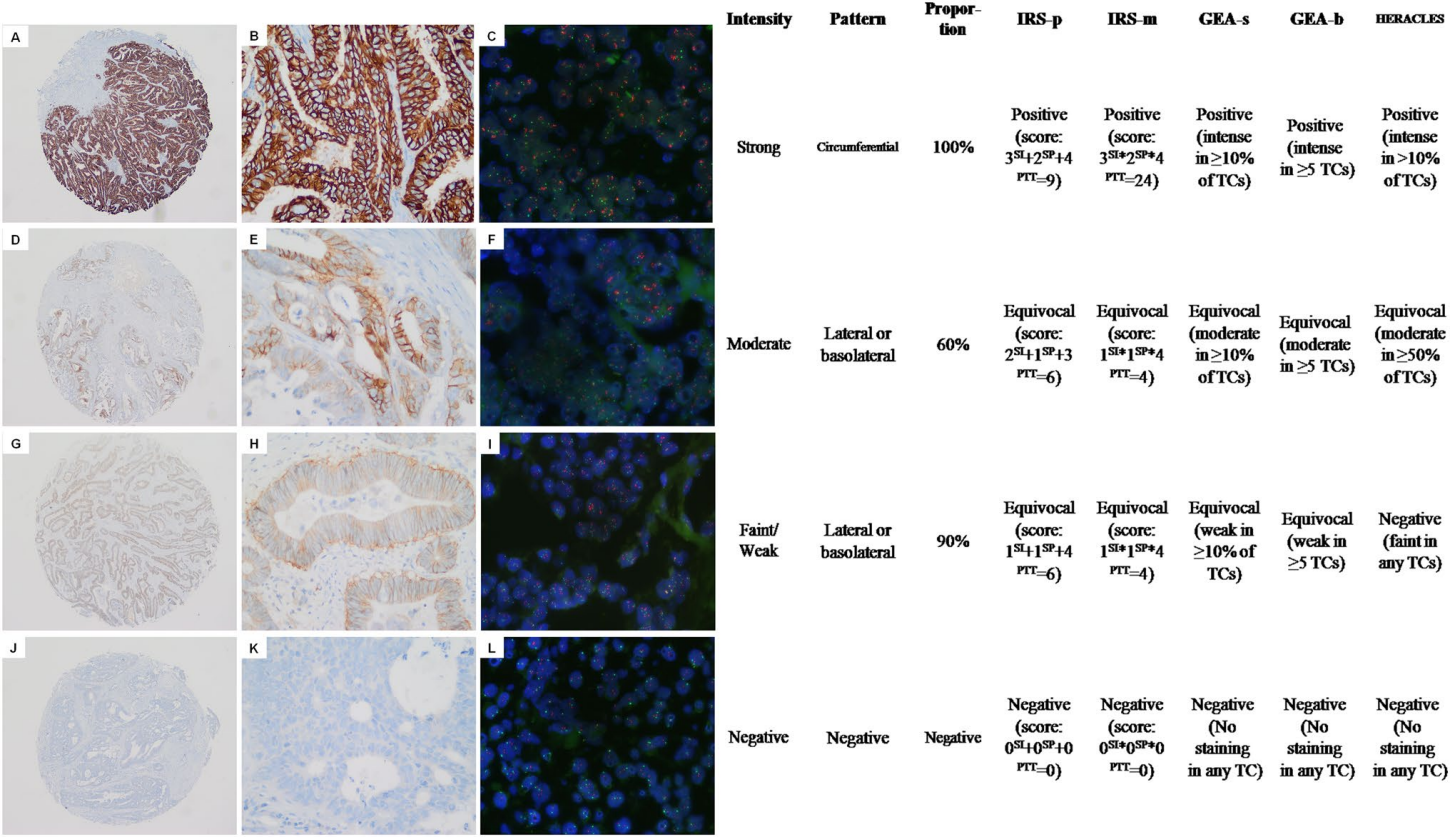
HER2 evaluation using five immunohistochemistry (IHC) assessment methods in TMA of colorectal cancer and their FISH test results. Representative immunostaining pattern (SP), intensity (SI) of tumor cells, and their percentage of positivity (PPT): **A** shows a case with a strong IHC cell membrane circumferential staining and its local magnification (**B**), FISH test (**C**) and corresponding scoring results; **D** shows a case with moderate lateral and basolateral staining and its corresponding local magnification (**E**), FISH test (**F**) and scoring results; **G** shows a case with faint/weak lateral or basolateral staining and its corresponding local magnification (**H**), FISH test (**I**) and scoring results; and **J** shows a negative staining case and its corresponding local magnification (**HK**), FISH test (**L**) and scoring results

In this study, HER2 immunoreactivity was presented with cell membrane staining pattern (SP), intensity (SI), and percentage of positive tumor cells (PPT) in TMAs. The SPs of HER2 on tumor cell membranes were classified into groups from 0 to 2 as follows: 0 (no staining), 1 (lateral or basolateral staining), and 2 (circumferential staining) (Fig. 1). The SI was categorized as follows: 0 (negative), 1(weak), 2 (moderate), or 3 (strong) (Fig. 1). The PPT were similarly subdivided as follows: 0 (< 5% expression), 1 (5 to 24% expression), 2 (25 to 49% expression), 3 (50 to 74% expression) and 4 (≥ 75% expression). Two kinds of immune response scores (IRS) were used to evaluate the expression of HER2, including the IRS-plus system (IRS-p), which added the scores of SP, SI, and PPT; and the IRS-multiply system (IRS-m) multiplied the scores of SP, SI, and PPT. Therefore, the IRS-p ranges from 0 to 9, while the IRS-m ranges from 0 to 24. Then, both IRS-p and IRS-m were classified into IHC 1 + (IRS-p score 1–2, IRS-m score 1–3), IHC 2 + (IRS-p score 3–7, IRS-m score 4–8), and IHC 3 + (IRS-p score 8–9, IRS-m score 12–24). IHC scores of 0 and 1 + were considered as being “HER2 negative”, IHC score of 2 + was considered as being “HER2 equivocal”, and IHC score of 3 + was considered as being “HER2-positive”.

The HERACLES criterion (Valtorta et al. 2015) and the GEA criterion [including scoring systems for both biopsy (GEA-b) and surgical (GEA-s) specimen] (Ruschoff et al. 2012) were also used to evaluate the specimens as previously described. The definition of the five IHC scoring systems described above is summarized in Table 1.

**Table 1:** Summary of immunohistochemical assessment methods for HER2

HER2 score	IHC classification	IRS-p	IRS-m	GEA-s	GEA-b	HERACLES
0	Negative	IRS-p score^1^ of 0	IRS-m score^2^ of 0	No staining or staining in < 10% of TCs	No staining in any TC	No staining, moderate staining in < 50% of TCs or intense staining in ≤ 10% of TCs
1 +	Negative	IRS-p scores range from 1 to 2	IRS-m scores range from 1 to 3	Faint staining in ≥ 10% of TCs	Faint staining in at least 1 cluster of ≥ 5 TCs	Faint staining in any TCs
2 +	Equivocal	IRS-p scores range from 3 to 7	IRS-m scores range from 4 to 8	Weak to moderate staining in ≥ 10% of TCs	Weak to moderate staining in at least 1 cluster of ≥ 5 TCs	Moderate staining in ≥ 50% of TCs
3 +	Positive	IRS-p scores range from 8 to 9	IRS-m scores range from 12 to 24	Intense staining in ≥ 10% of TCs	Intense staining in at least 1 cluster of ≥ 5 TCs	Intense staining in > 10% of TCs

*HER2* human epidermal growth factor receptor 2, *IHC* immunohistochemistry

^1^ The IRS-p score was calculated by summing the scores of immunohistochemical staining pattern, intensity and positive ratio. ^2^ the IRS-m score was calculated by multiplying the scores of immunohistochemical staining pattern, intensity and positive ratio

### FISH

**Fig. 2:**
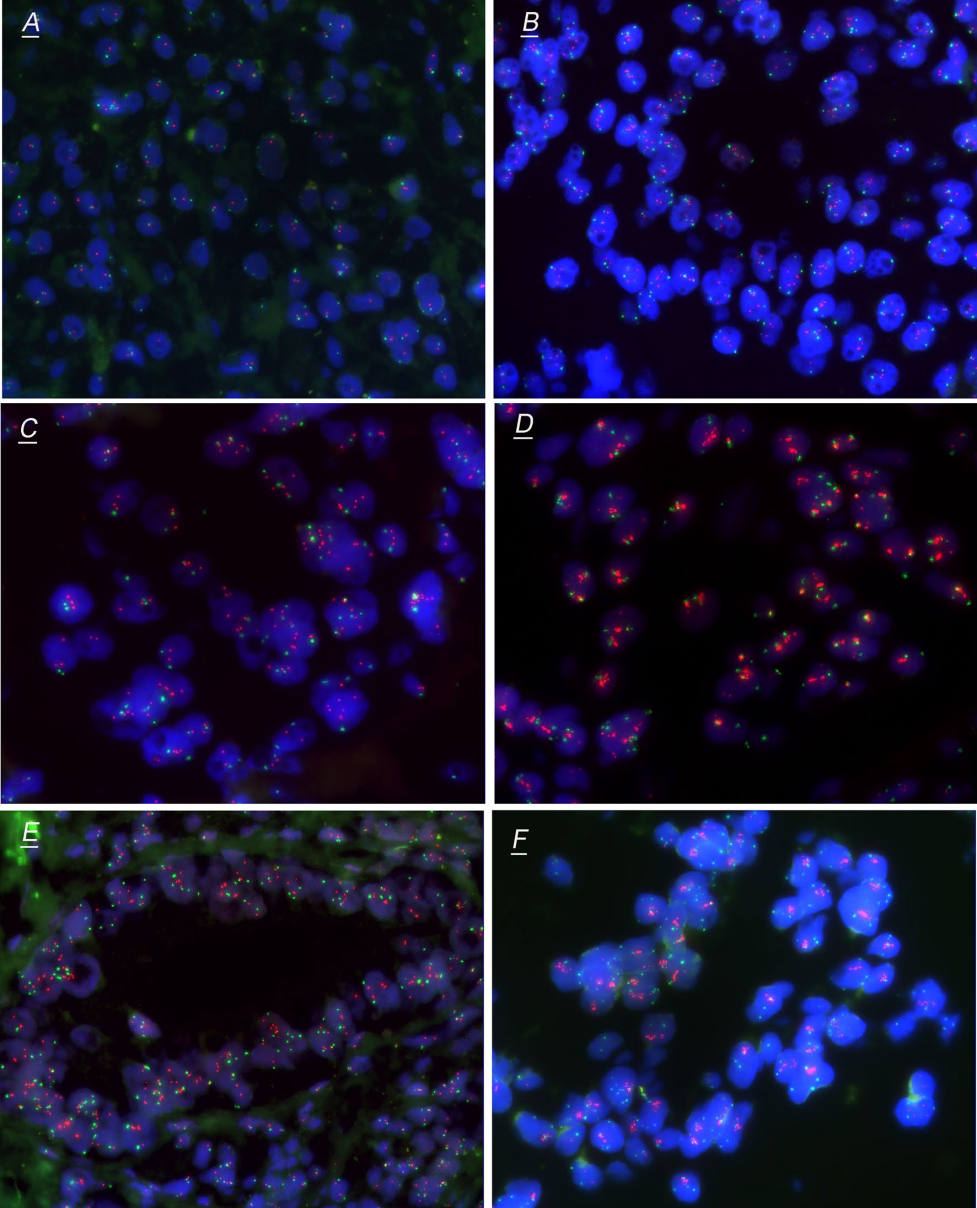
HER2 evaluation by fluorescence in situ hybridization (FISH) in TMA of colorectal cancer. Representative FISH pattern of tumor cells HER2/CEP17 < 2.0 and HER2cn < 4.0 (**A**), Her2/CEP17 < 2.0 and 4.0 ≤ HER2cn < 6.0 (**B**), HER2/CEP17 ≥ 2.0 meanwhile HER2cn < 4.0 (**C**), HER2/CEP17 ≥ 2.0 meanwhile 4.0 ≤ HER2cn < 6.0 (**D**), HER2/CEP17 ≥ 2.0 meanwhile HER2cn ≥ 6.0 (**E**) and Her2/CEP17 < 2.0 meanwhile HER2cn ≥ 6.0 (**F**). **A**, **B** were classified into FISH group 1 and group 2, respectively. **C**, **D** were classified into FISH group 3, and **E**, **F** were classified into FISH group 4

Commercially available, locus-specific HER2 probe (190-kb Spectrum Orange directly labeled fluorescent DNA probe) and CEP17 probe (5.4-kb Spectrum Green directly labeled fluorescent DNA) were used according to the manufacturer’s recommendations (Jinpujia, Beijing, China). No less than 20 non-overlapping nuclei of tumor cells per sample were evaluated for HER2 probe (red) and CEP17 probe (green) signals, and the signal counting results of HER2 and CEP17 were recorded for further evaluation. We further divided all the samples into four groups according to the FISH results: FISH group 1 [i.e., HER2/CEP17 < 2.0 and average HER2 copy number (HER2cn) < 4.0], FISH group 2 (i.e., Her2/CEP17 < 2.0 and 4.0 ≤ HER2cn < 6.0), FISH group 3 (i.e., HER2/CEP17 ≥ 2.0 and HER2cn < 6.0), and FISH group 4 (i.e., HER2cn ≥ 6.0). FISH group 3 including HER2/CEP17 ≥ 2.0 meanwhile HER2cn < 4.0 and HER2/CEP17 ≥ 2.0 meanwhile 4.0 ≤ HER2cn < 6.0. FISH group 4 including HER2/CEP17 ≥ 2.0 meanwhile ≥ 6.0 and Her2/CEP17 < 2.0 meanwhile ≥ 6.0 (Fig. 2). Since there were very few cases of HER2/CEP17 ≥ 2.0 meanwhile HER2cn < 4.0, we classified them into group 3 rather than as an independent group.

### Analysis

IHC staining for mismatch repair (MMR) protein was performed to assess MMR deficiency. MMR-deficient (dMMR) was defined as the absence of MLH1, MSH2, MSH6, or PMS2 expression in the nuclei of tumor cells, with infiltrating lymphocytes as the internal positive control. In contrast, positive nuclear staining for all four MMR proteins was classified as MMR-proficient (pMMR).

HER2 immunostaining was scored by two independent pathologists (QS and FP G) without knowing the clinicopathological parameters in advance. Discrepancies between the observers were resolved through discussion. If there is heterogeneity in IHC staining between the two core samples for each tumor, the whole-slide was stained for final decision. FISH result was used as the golden standard and cutoff for HER2 IHC positivity. Receiver-operating characteristic (ROC) curves were used to determine the test performance of each HER2 IHC scoring method.

### Statistical analysis

Distributed data of continuous variables were represented as “mean ± standard deviation” and “median (range)”. Analysis of variance or the Kruskal–Wallis rank-sum test was used to compare differences among groups. The Chi-square or Fisher's exact test was utilized to compare the ratios. Patient post-resection survival was estimated by the Kaplan–Meier method with a log-rank test. Statistical analysis was performed with SPSS 19.0 software (SPSS Inc, Chicago, Illinois, US). Differences were considered statistically significant when the *p* value was less than 0.05.

## Results

### Clinicopathological characteristics

A total of 664 CRC cases were included in this study, of which there were 387 (58.3%) males and 277 (41.7%) females. The mean age was 61.4 years (range 22–89 years). CRC occurred in the right-sided colon in 400 (60.2%) cases and in the left-side colon in 264 (39.8%) cases. 65 CRCs were classified as dMMR, and the remaining 599 cases were pMMR. Follow-up data were available in 90.4% (600/664) of the patients, and the median follow-up period for the survival analyses was 23 months (range 5–77 months).

### Correlation of FISH subgroups with clinicopathological and prognostic features

**Fig. 3:**
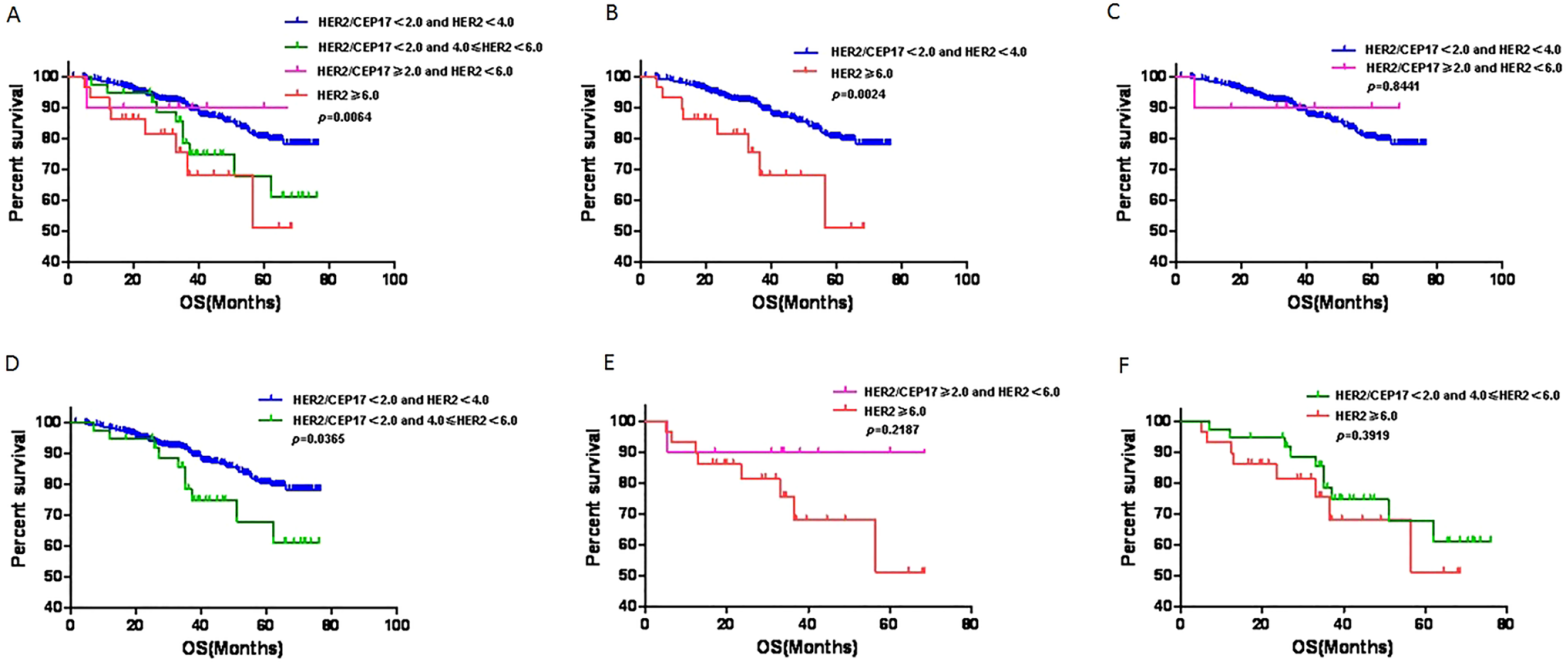
Kaplan–Meier curves of overall survival among FISH subgroups (**A**), HER2/CEP17 < 2.0 and HER2cn < 4.0 vs. HER2cn ≥ 6.0 (**B**), HER2/CEP17 < 2.0 and HER2cn < 4.0 vs. HER2/CEP17 ≥ 2.0 and HER2cn < 6.0 (**C**), HER2/CEP17 < 2.0 and HER2cn < 4.0 vs. Her2/CEP17 < 2.0 and 4.0 ≤ HER2cn < 6.0 (**D**), HER2/CEP17 ≥ 2.0 and HER2cn < 6.0 vs. HER2cn ≥ 6.0 (**E**), and Her2/CEP17 < 2.0 and 4.0 ≤ HER2cn < 6.0 vs. HER2cn ≥ 6.0 (**F**). The log-rank test was used to calculate the *P* value

Based on the HER2 scoring system for gastric (Ruschoff et al. 2012) and breast cancer (Rakha et al. 2015), FISH HER2-positive status was defined as HER2/CEP17 ≥ 2.0 or/and a mean HER2cn ≥ 6.0. In this study, we further divided the samples into four groups according to the FISH results: FISH group 1 (i.e., HER2/CEP17 < 2.0 and HER2cn < 4.0), FISH group 2 (i.e., Her2/CEP17 < 2.0 and 4.0 ≤ HER2cn < 6.0), FISH group 3 (i.e., HER2/CEP17 ≥ 2.0 and HER2cn < 6.0), and FISH group 4 (i.e., HER2cn ≥ 6.0). FISH group 4 including HER2/CEP17 ≥ 2.0 meanwhile ≥ 6.0 and Her2/CEP17 < 2.0 meanwhile ≥ 6.0 (Fig. 2). First, we assessed the clinicopathological characteristics of the four FISH scoring groups (Table 2). Tumor invasion depth (pT stage) (*p* = 0.031), pN stage (*p* = 0.009), and OS (*p* = 0.006) (Fig. 3) were significantly different among the four FISH groups. Whereas, only the FISH group 4 had higher pT stage (*p* = 0.004), pN stage (*p* = 0.015), and worse OS (*p* = 0.002) than that of the FISH group 1. Except for the significant differences in MMR status (*p* = 0.026) and OS (*p* = 0.037) between FISH group 1 and 2, there were no significant differences in the comparison of clinicopathological features and prognosis between the remaining groups. Therefore, we regarded group 1 and group 4 represent two significant subtypes: group 1 represented the typical amplification-negative cases and group 4 represented the typical amplification-positive cases. As to group 2 and 3, they cannot be divided into independent subtypes. When HER2/CEP17 ratio ≥ 2.0 and average HER2cn ≥ 6.0 were used as thresholds, the positive rates of HER2 amplification were 6.63% (44/664) and 4.97% (33/664), respectively. However, no matter for gastric or breast cancer, HER2/CEP17 ≥ 2.0 both regarded as FISH HER2 positive, so we also set HER2/CEP17 ≥ 2.0 or a mean HER2cn ≥ 6.0 as FISH amplification standard. According to the standard above, the amplification rate of HER2 gene in this study was 7.08% (47/664).

**Table 2:** Clinicopathological features of different CRC FISH score subgroups

Variables	HER2/CEP17 <2.0 and HER2cn <4.0 Group 1 *N* = 577	HER2/CEP17 < 2.0 and 4.0 ≤ HER2cn < 6.0 Group 2 *N* = 40	HER2/CEP17 ≥ 2.0 and HER2cn < 6.0 Group 3 *N* = 14	HER2cn ≥ 6.0 Group 4 *N* = 33	*p*^1^ value	*p*^2^ value	*p*^3^ value	*p*^4^ value	*p*^5^ value	*p*^6^ value
Gender (male/female)	340/237	23/17	9/5	15/18	0.466	0.147	0.788	0.869	0.341	0.352
Age (mean)	61.24(25–89)	61.81(47–84)	61.26(48–79)	61.73(22–83)	1.000	0.968	0.936	0.856	0.864	0.952
Tumor location Right-sided colon Left-sided colon	351 226	23 17	8 6	18 15	0.870	0.471	0.788	0.739	1.000	0.817
Tumor size (mean ± SD)	2.68 (0.7–6)	2.54 (0.5–8)	2.52(0.1–9)	2.32 (0.4–9)	0.963	0.563	0.642	0.952	0.930	0.830
Differentiation (well/moderate/poor)	22/524/31	0/40/0	0/13/1	1/30/2	0.579	0.962	0.737	0.134	0.800	0.150
LVI (yes/no)	185/392	12/28	5/9	17/16	0.307	0.197	1.000	0.312	0.358	0.092
PI (yes/no)	268/309	23/17	6/8	16/17	0.581	0.859	1.000	0.193	0.760	0.486
pTNM stage (I/II/III/IV))	56/204/299/18	4/13/19/4	1/7/5/1	1/8/21/3	0.174	0.084	0.514	0.158	0.277	0.460
pT stage (T1/T2/T3/T4)	13/59/469/36	1/3/34/2	0/1/10/3	1/0/25/7	0.031*	0.004*	0.146	0.931	0.422	0.088
pN stage (N0/N1/N2)	261/216/100	17/9/14	8/3/3	9/12/12	0.009*	0.015*	0.472	0.013	0.150	0.302
DM (M0/M1)	559/18	37/3	13/1	30/3	0.156	0.099	0.370	0.149	1.000	1.000
MMR status (pMMR/dMMR)	514/63	40/0	14/0	31/2	0.065	0.563	0.382	0.026*	1.000	0.201

*FISH* fluorescent in situ hybridization, *LVI* lymphatic vascular invasion, *PI* perineural invasion, *DM* distant metastasis, *MMR* mismatch repair

^1^Represents comparison among four different FISH subgroups (group 1–4). ^2^Represents the comparison between group 1 and group 4. ^3^Represents the comparison between group 1 and group 3. ^4^Represents the comparison between group 1 and group 2. ^5^Represents the comparison between group 3 and group 4. ^6^Represents the comparison between group 2 and group 4. *Statistically significant, *p* < 0.05

### IRS-p is more suitable as the IHC scoring criteria for CRC

**Fig. 4:**
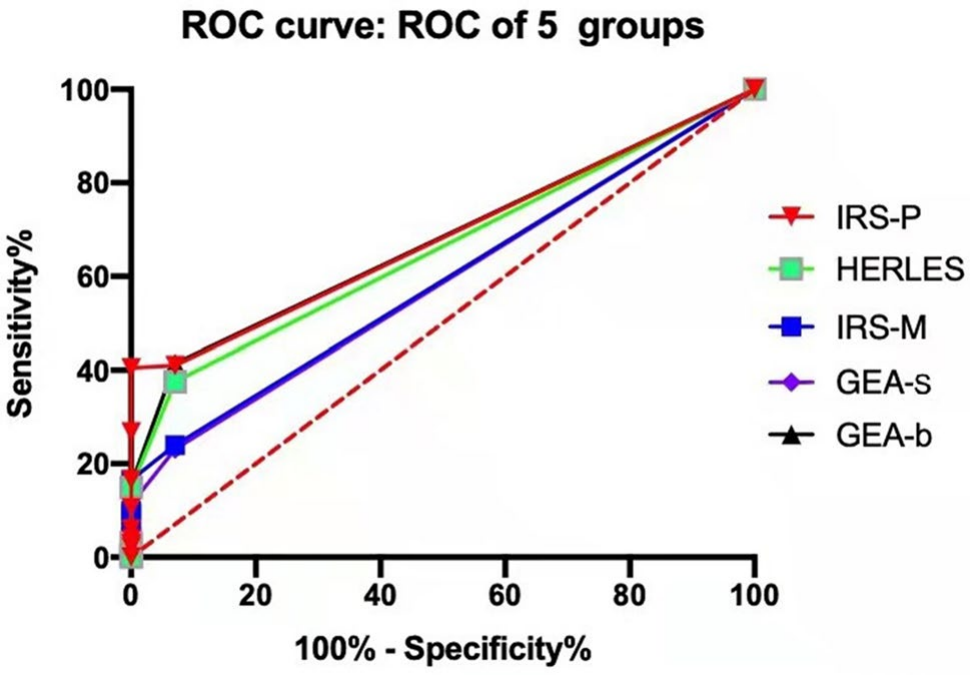
Receiver operator characteristic curve plotting test sensitivity in relation to specificity between immunohistochemical scoring criteria of IRS-p, IRS-m, GEA-s, GEA-b and HERACLES

According to the IHC scoring criteria of IRS-p, IRS-m, GEA-s, GEA-b and HERACLES, the positive expression rates of HER2 (IHC 3 +) were 2.71%, 3.16%, 2.56%, 2.71% and 3.16%, respectively. The comparison of HER2 expression and FISH results in 664 CRCs using different IHC scoring criteria is presented in Table 3. The areas under the ROC curve of five IHC scoring criteria, namely IRS-p, IRS-m, GEA-s, GEA-b and HERACLES, were 0.6838, 0.5902, 0.5842, 0.6771 and 0.6577, respectively (Fig. 4). The results showed that IRS-p was more sensitive and specific than other IHC scoring systems.

**Table 3:** Comparison of different HER2 IHC scoring systems with FISH results

Groups	IRS-p		IRS-m		GEA-s		GEA-b		HERACLES	
	IHC	FISH positive (%)	IHC	FISH positive (%)	IHC	FISH positive (%)	IHC	FISH positive (%)	IHC	FISH positive (%)
0	392	9(2.30%)	505	15(2.97%)	511	15(2.94%)	390	9(2.31%)	415	11(2.65%)
1 +	94	2(2.13%)	98	6(6.12%)	78	2(2.56%)	171	5(2.92%)	150	3(2.0%)
2 +	160	19(11.88%)	40	8(20.0%)	58	14(24.14%)	85	16(18.82%)	78	15(19.23%)
3 +	18	17(94.44%)	21	18(85.71%)	17	16(94.18%)	18	17(94.44%)	21	18(85.71%)
All	664	47(7.08%)	664	47(7.08%)	664	47(7.08%)	664	47(7.08%)	664	47(7.08%)

*HER2* human epidermal growth factor receptor 2, *IHC* immunohistochemistry, *FISH* fluorescent in situ hybridization

In addition, no matter which IHC scoring system was taken, there were false-negative cases in the groups evaluated as 0 and 1 + . Taking IRS-p as an example, set FISH as the golden standard, the false-negative rates were 2.30% (9/392) and 2.13% (2/94) in 0 and 1 + groups, respectively (Table 3). In the HERACLES system, faint/weak expression of HER2 on the membrane of any proportion of tumor cells (TCs) is interpreted as negative. However, in this study, there were 2 cases in which > 90% of the TCs weakly expressed HER2, which was eventually confirmed by FISH as HER2 amplification (Fig. 1G), so focusing only on staining intensity may lead to a small number of HER2-positive cases being missed. Therefore, if we take IHC as the screening method for target therapy, IHC 0 or 1 + cases are still needed further FISH test to confirm HER2 gene status. In our study, only one case with IRS-p IHC 3 + and negative FISH showed a false positive for IHC. In this case, FISH was further performed on the whole slide; however, the result still showed the same as the TMA’s.

### Correlation of IRS-p subgroups with clinicopathological and prognostic features

**Fig. 5:**
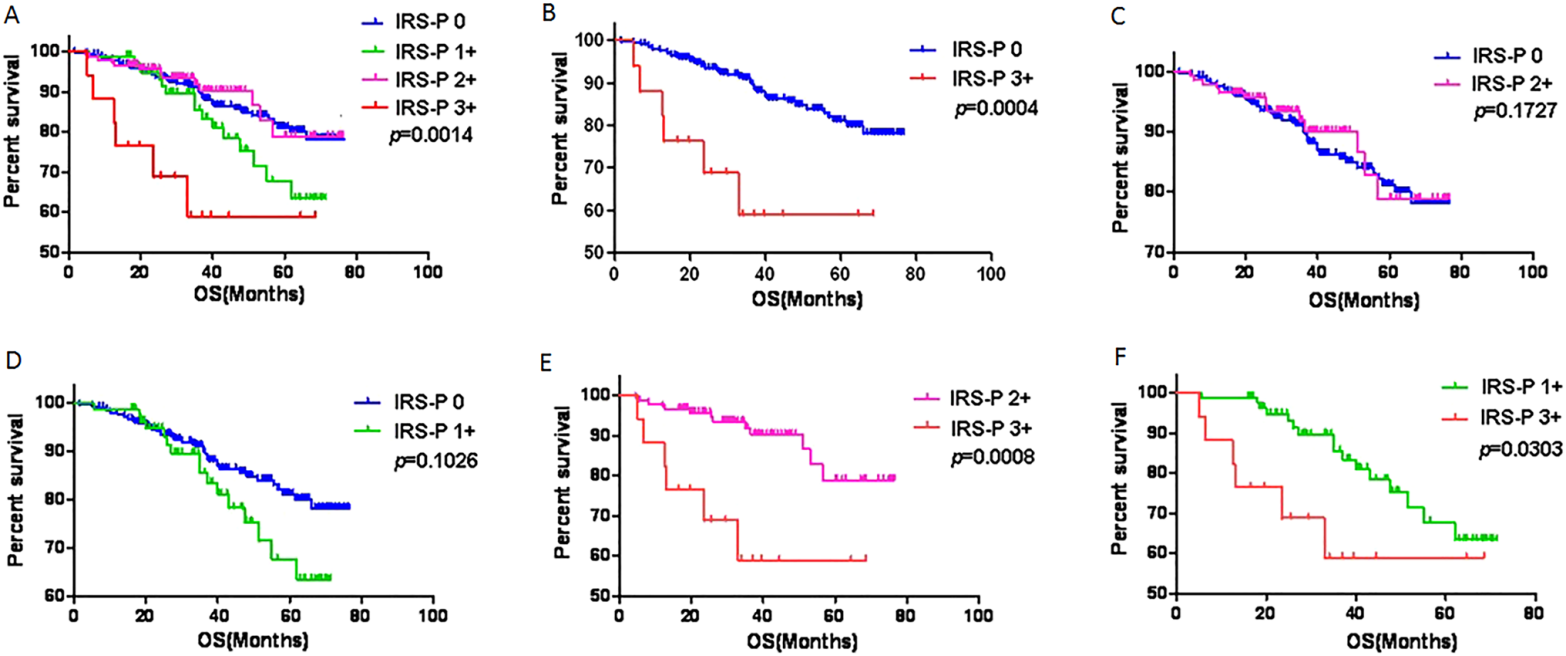
Kaplan–Meier curves of overall survival among IRS-p score 0–3 + (**A**), 0 vs. 3 + (**B**), 0 vs. 2 + (**C**), 0 vs. 1 + (**D**), 2 + vs. 3 + (**E**), and 1 + vs. 3 + (**F**). The log-rank test was used to calculate the *P* value

The clinicopathological features of HER2 IHC 0–3 + CRCs (IRS-p group 1–4) using the IRS-p scoring system are summarized in Table 4. There are significant difference in tumor differentiation (*p* = 0.038), lymphatic vascular invasion (LVI) (*p* = 0.001), lymph node metastasis (pN stage) (*p* = 0.043) and OS (*p* <  0.001) among IHC 0–3 + groups. Further subgroup analysis revealed a more aggressive biologic behavior in the IHC 3 + CRCs (IRS-p group 4). Compared with the IHC 0 group (IRS-p group 1), the IHC3 + group had more frequent LVI (*p* = 0.044), higher AJCC tumor stage (pTNM stage) (*p* = 0.044) and pN stage (*p* = 0.007), and worse OS (*p* = 0.004) (Fig. 5). There were no significant differences in clinicopathological features between the IHC 1 + (IRS-p group 2) and IHC 0 groups, whereas there were significant differences between the IHC 1 + and IHC 3 + groups in terms of tumor differentiation (*p* = 0.039), LVI (*p* = 0.033), pN stage (*p* = 0.020) and OS (*p* = 0.030). Therefore, the IHC 1 + and IHC 0 groups, both of which are considered as HER2 negative, have similarities in clinicopathological features and prognosis. In the same way, we compared the IHC 2 + group (IRS-p group 3) with the IHC 0 and IHC 3 + groups, respectively. When compared IHC 2 + group with IHC 0 group, there were significant differences in tumor differentiation (*p* = 0.022) and LVI (*p* = 0.002); and there were also significant differences in LVI (*p* = 0.001), pTNM stage (*p* = 0.027) and pN stage (*p* = 0.001) between IHC 2 + group and IHC 3 + group. Thus, IHC 2 + CRCs, as a state of HER2-equivocal amplification, are a distinct subgroup that differs significantly from IHC 0 or3 + CRCs in clinicopathologic characteristics.

**Table 4:** Clinicopathological characteristic of different CRC IRS-p score subgroups

Variables	IHC 0 Group 1, *N* = 392	IHC 1 + Group 2, *N* = 94	IHC 2 + Group 3, N = 160	IHC 3 + Group 4, *N* = 18	*p*^1^ value	*p*^2^ value	*p*^3^ value	*p*^4^ value	*p*^5^ value	*p*^6^ value
Gender (male/female)	234/158	57/37	86/74	10/8	0.584	0.808	0.217	0.907	1.000	0.794
Age (mean)	61.3(25–86)	61.2(28–84)	61.2(27–89)	61.8(22–83)	1.000	1.000	1.000	1.000	1.000	1.000
Tumor location Right-sided colon Left-sided colon	240 152	54 40	93 67	13 5	0.674	0.460	0.504	0.577	0.315	0.300
Tumor size (mean ± SD)	2.67 (0.2–11)	2.95 (0.4–9)	2.33 (0.5–8)	2.56(0.7–6)	0.963	0.987	0.632	0.658	0.836	0.952
Differentiation (well/moderate/poor)	9/357/26	4/89/1	10/145/5	0/16/2	0.038*	0.629	0.022*	0.066	0.153	0.039*
LVI (yes/no)	141/251	31/63	36/124	11/7	0.001*	0.044*	0.002*	0.632	0.001*	0.033*
PI (yes/no)	184/208	48/46	73/87	8/10	0.853	1.000	0.851	0.492	1.000	0.798
pTNM stage (I/II/III/IV)	33/143/203/13	10/29/48/7	19/58/78/5	0/2/15/1	0.105	0.044*	0.646	0.236	0.027*	0.075
pT stage (T1/T2/T3/T4)	9/37/318/28	1/12/76/5	5/14/129/12	0/0/15/3	0.624	0.242	0.942	0.623	0.288	0.156
pN stage (N0/N1/N2)	176/139/77	40/35/19	77/59/24	2/8/8	0.043*	0.007*	0.436	0.917	0.001*	0.020*
DM (M_0_/M_1_)	378/14	89/5	155/5	17/1	0.799	0.496	1.000	0.387	0.478	1.000
MMR status (pMMR/dMMR)	359/33	83/11	140/20	17/1	0.404	1.000	0.153	0.320	0.700	0.687

*IHC* immunohistochemistry, *LVI* lymphatic vascular invasion, *PI* perineural invasion, *DM* distant metastasis, *MMR* mismatch repair

^1^Represents comparison among IHC Score 0–3 + subgroups (group 1–4). ^2^Represents the comparison between group 1 and group 4. ^3^Represents the comparison between group 1 and group 3. ^4^Represents the comparison between group 1 and group 2. ^5^Represents the comparison between group 3 and group 4. ^6^Represents the comparison between group 2 and group 4. *Statistically significant, *p* < 0.05

## Discussion

Nowadays, HER2 has emerged as an important therapeutic target and prognostic factor for both primary and metastatic CRC (Sawada et al. 2018; Richman et al. 2016). However, neither the scoring criteria for HER2 IHC and FISH assessment nor the clinicopathological features of different HER2 statuses have reached a consensus in CRC. The positivity of HER2 expression (including membranous and cytoplasmic expression) in CRC ranged from 1.3 to 82% owing to different detection methods and scoring systems (Richman et al. 2016; McKay et al. 2002; Blok et al. 2013). In 2015, Valtorta et al. (2015) developed the HERACLES diagnostic criterion for HER2 positivity in CRC, which has been a wildly acceptable scoring system for HER2 assessment. Besides the HERACLES criterion, the GEA criteria have also been used for HER2 assessment in CRC (Liu et al. 2020). The HER2 assessment methods for surgical specimens (GEA-s) and biopsy specimens (GEA-b) are slightly different in GEA system, which are briefly described as follows (Bartley et al. 2017): More than 10% of tumor cells in surgical specimens and ≥ 5 tumor cells in biopsy specimens showed HER2 IHC 3 + score, or HER2 IHC 2 + score was detected in ≥ 10% of tumor cells in surgical specimens and in ≥ 5 tumor cells in biopsy specimens, while FISH HER2/CEP17 ≥ 2.0. In this study, we performed HER2 IHC and FISH in a large Chinese cohort of 664 CRCs, and tested the validity of 5 IHC scoring criteria (i.e., IRS-p, IRS-m, GEA-s, GEA-b and HERACLES) using FISH as the threshold for confirming HER2 amplification. Finally, the sensitivity and specificity of IRS-p in judging HER2 overexpression/amplification are superior to those of other scoring systems.

Liu et al. (2020) analyzed the correlation between HER2 positivity and clinicopathological features of CRC according to the HERACLES and GEA criteria, respectively. They found that HER2-positive CRC diagnosed by HERACLES criterion was associated with left-side colon location and a higher pN and pTNM stage, whereas no correlation between HER2 positivity and clinicopathological features was shown according to GEA criterion. We investigated the correlation between the IRS-p HER2 immune scores and clinicopathological features of CRC. The result showed a significant difference in tumor differentiation, LVI, pN stage and OS among IHC 0–3 + groups. Intergroup comparison showed that the clinicopathological features of IHC 1 + group were similar to those of IHC 0 (HER2-negative) group and significantly different from those of IHC 3 + (HER2-positive) group in terms of LVI, pN stage and OS. In contrast, the IHC 2 + (HER2-equivocal) group was distinct from the HER2-positive and -negative groups. These results suggest that the HER2 immune score based on IRS-p criteria can classify cases with different clinicopathological features appropriately. Therefore, we consider that the IRS-p scoring system, which superimposes the scores of IHC staining patterns, intensities, and proportions, is more suitable for CRC.

HER2 overexpression ratio was low in our cohort. According to the IHC scoring criteria of IRS-p, IRS-m, GEA-s, GEA-b and HERACLES, the positive expression rates of HER2 (IHC 3 +) were 2.71%, 3.16%, 2.56%, 2.71% and 3.16%, respectively. In a large cohort that pooled 3256 CRCs from three clinical trials (i.e., QUASAR, FOCUS and PICCOLO) based on a Western population, HER2 overexpression occurred in 1.3% (25/1914) of stage II–III CRCs and 2.2% (29/1342) of stage IV CRCs (Richman et al. 2016). In another study from China, the authors used the HERACLES criteria and the GEA criteria to assess the HER2 positivity in CRCs, which was 2.6% and 2.9%, respectively (Liu et al. 2020). The incidence of HER2 positivity in these studies is consistent with our findings, suggesting that HER2 overexpression remains a small probability molecular event during the malignant process of CRC.

Using FISH as the definitive criterion for HER2 amplification status, the HER2 positivity rate in this study was 7.08%, which was generally consistent with the HER2 amplification rate reported by TCGA (Cancer Genome Atlas N 2012). However, when referring to HER2cn ≥ 6.0 as the threshold, the positive rate of HER2 was 4.97%, and when taking HER2/CEP17 ≥ 2.0 as the threshold, the positive rate of HER2 was 6.63%. Thus, even though FISH is recognized as the gold standard for HER2 amplification, different threshold choices can lead to different positive rates. Since the evaluation criteria of HER2 have not been standardized in CRC and have varied from one study to another, different detection methods, antibodies and evaluation systems used to define HER2 overexpression/amplification may contribute to different HER2 positivity rates (Richman et al. 2016; McKay et al. 2002; Blok et al. 2013; Liu et al. 2020; Lee et al. 2014).

In the present study, the FISH results were consistent with the IHC results in the great majority of cases, but there were still a minimal number of cases determined to be HER2 negative by IHC (IHC 0 and 1 +) showed HER2 gene amplification in FISH. The same phenomenon occurs in 1.5–6% of breast cancers (Gibbons-Fideler et al. 2019; Ellis et al. 2005) and 2.1–4% of gastric cancers (Kim et al. 2007; Tafe et al. 2011; Hofmann et al. 2008; Robertson et al. 2018), and is attributed to HER2 intra-tumoral heterogeneity, co-amplified/polysomy CEP17 and monosomy CEP17, and so on (Gibbons-Fideler et al. 2019; Robertson et al. 2018). In this circumstance, even the IHC score 0 or 1 + but with FISH positive could be defined as HER2 positive and may benefit from HER2-based target treatment (Gibbons-Fideler et al. 2019). However, the published national comprehensive cancer network recommends using IHC as the frontline test and subjecting only IHC 2 + equivocal samples for FISH analysis (Valtorta et al. 2015). Therefore, we recommend combined IHC and FISH testing for CRC patients who intend to undergo HER2-targeted therapy. The heterogeneity of HER2 status is usually manifested by two conditions, one is the heterogeneity between the local and the tumor as a whole, which often represents the difference between biopsy specimens and the whole tumor sections in clinical setting; the other is the heterogeneity between the DNA level and protein level of HER2 status, which often represents the difference between FISH and IHC results. Besides DNA amplification, there are other mechanisms that induce high-expression of HER2 protein, including activation of HER2 transcript levels and mutations in its kinase domain (Downs-Kelly et al. 2005; Cocco et al. 2019; Pahuja et al. 2018). This may explain the IHC 3 + while FISH-negative situation.

According to the FISH results, the samples were further divided into four subgroups in this study, including two negative groups (group 1 with HER2/CEP17 < 2.0 and HER2cn <  4.0, group 2 with HER2/CEP17 <  2.0 and 4.0 ≤ HER2cn < 6.0) and two positive groups (group 3 with HER2/CEP17 ≥ 2.0 and HER2cn < 6.0, group 4 with HER2cn ≥ 6.0). Only group 1 and 4 showed significant differences in pT stage, pN stage and OS. Cases with HER2/CEP17 < 2.0 and HER2cn ≥ 4.0 but < 6.0 and cases with HER2/CEP17 ≥ 2.0 and HER2cn < 6.0 serve as independent FISH subgroups in breast cancer with distinct clinicopathological features (Yang et al. 2020). However, in our study, there were no significant differences in clinicopathological features and prognosis between groups 2, group 3 and the remaining groups, except for differences in MMR status and OS between groups 1 and 2. In addition, due to the relatively small sample size in groups 2 and 3, the clinicopathological characteristics of CRC with different HER2 FISH status and their impact on patient survival still need to be further validated on a larger scale. But in this study, HER2 overexpression or gene amplification had a negative impact on the prognosis of CRC patients.

This study is limited using TMA methodology to detect HER2 status. Although two tumor cores were derived from representative regions of each CRC and necrotic tissue was avoided, the heterogeneity of HER2 overexpression/amplification within the tumor was inevitable. Therefore, there may be bias in the analysis of the correlation between HER2 status and the clinicopathological features and prognosis of CRC patients. However, given our use of FISH results as the threshold for HER2 status, several previous studies have shown that FISH results from TMAs (Kunz et al. 2012) or biopsies (Grillo et al. 2013) and whole sections are largely comparable. In addition, as a result of a relatively high rate of loss to follow-up (9.64%, 64/664), we did not evaluate OS in the entire cohort. Finally, this was a retrospective, descriptive study, and we did not investigate the effects of HER2-targeted therapy in CRC patients. Nowadays, many clinical investigations have been conducted with single or combined HER2 inhibition such as Trastuzumab, lapatinib or pertuzumab. All of these trials often based on specific HER2 amplification or high-expression criteria. For example, HERACLES-A phase II trial using HERACLES criteria to recruit HER2-amplified mCRC to assess the activity of trastuzumab combined with lapatinib. However, the limitations in our study is no access to response rates based on our criteria (no matter FISH or IHC) or no related clinical HER2 trials using our criteria until now.

In conclusion, our study is the first to use five IHC scoring systems to assess HER2 expression and evaluate the efficacy of the five IHC scoring criteria with FISH results as the threshold for HER2 overexpression/amplification. The results showed that the IRS-p criterion was more suitable than other IHC criteria, including the HERACLES and GEA criteria, for assessing HER2 status in CRC patients. Whereas for FISH scoring system, only HER2/CEP17 <  2.0 meanwhile HER2cn < 4.0 and HER2cn ≥ 6.0 were subgroups with unique clinicopathological characteristics.
